# Susceptible and Prognostic Genetic Factors Associated with Diabetic Peripheral Neuropathy: A Comprehensive Literature Review

**DOI:** 10.1155/2018/8641942

**Published:** 2018-03-15

**Authors:** L. B. L. Prabodha, N. D. Sirisena, V. H. W. Dissanayake

**Affiliations:** Human Genetics Unit, Faculty of Medicine, University of Colombo, Colombo, Sri Lanka

## Abstract

Type 2 diabetes mellitus (T2D) is a disorder of glucose metabolism. It is a complex process involving the regulation of insulin secretion, insulin sensitivity, gluconeogenesis, and glucose uptake at the cellular level. Diabetic peripheral neuropathy (DPN) is one of the debilitating complications that is present in approximately 50% of diabetic patients. It is the primary cause of diabetes-related hospital admissions and nontraumatic foot amputations. The pathogenesis of diabetic neuropathy is a complex process that involves hyperglycemia-induced oxidative stress and altered polyol metabolism that changes the nerve microvasculature, altered growth factor support, and deregulated lipid metabolism. Recent literature has reported that there are several heterogeneous groups of susceptible genetic loci which clearly contribute to the development of DPN. Several studies have reported that some patients with prediabetes develop neuropathic complications, whereas others demonstrated little evidence of neuropathy even after long-standing diabetes. There is emerging evidence that genetic factors may contribute to the development of DPN. This paper aims to provide an up-to-date review of the susceptible and prognostic genetic factors associated with DPN. An extensive survey of the scientific literature published in PubMed using the search terms “Diabetic peripheral neuropathy/genetics” and “genome-wide association study” was carried out, and the most recent and relevant literature were included in this review.

## 1. Introduction

**Figure 1:**
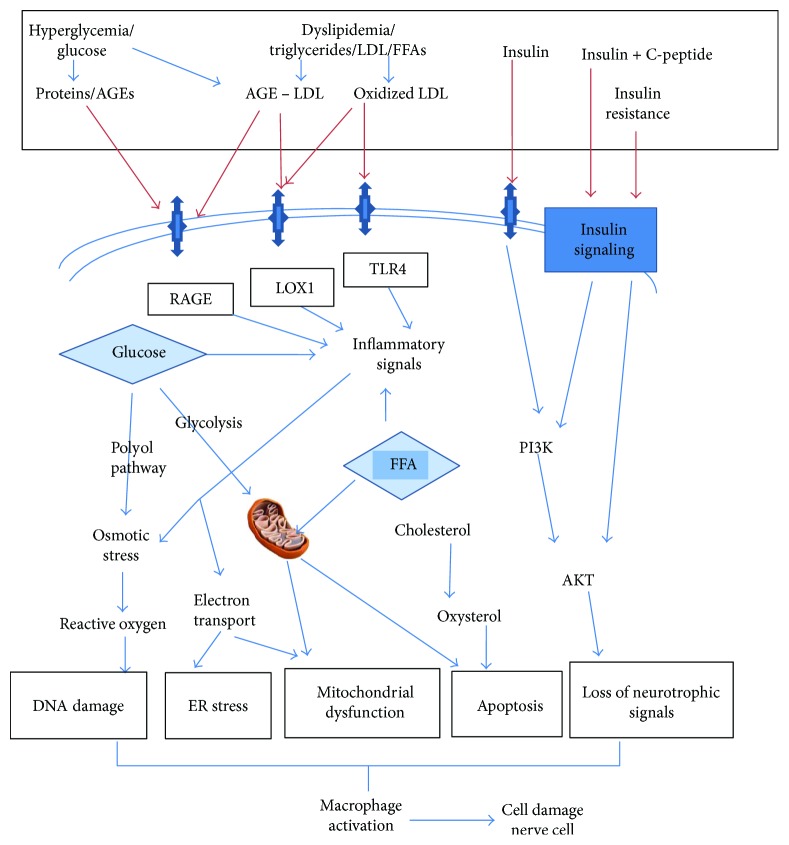
Mechanisms of diabetic neuropathy. Aetiological factors of diabetes initiate a cascade of events leading to DNA damage, endoplasmic reticulum stress, mitochondrial complex dysfunction, apoptosis, and loss of neurotrophic signaling. Ultimate activation of macrophages will cause cell damage in neurons, glial cells, and vascular endothelial cells, all of which can result in nerve dysfunction and neuropathy. AGE = advanced glycation end-products; LDL = low-density lipoprotein; FFA = free fatty acids; ER = endoplasmic reticulum; PI3K = phosphatidylinositol-3-kinase; LOX1 = oxidized LDL receptor 1; RAGE = receptor for advanced glycation end-products; TLR4 = Toll-like receptor 4.

Diabetes mellitus is nowadays one of the foremost noncommunicable diseases affecting more than 387 million people worldwide [1]. Type 2 diabetes mellitus (T2D) is a disorder of glucose metabolism. It is a complex process involving the regulation of insulin secretion, insulin sensitivity, gluconeogenesis, and glucose uptake at the cellular level. Dysregulation of one or more of these processes due to environmental or genetic factors can lead to altered glucose metabolism causing diabetes mellitus [2, 3]. More than 90% of cases of T2D show higher incidence of insulin resistance. This phenomenon is acquired due to sedentary lifestyle in combination with multifactorial genetic susceptibility. T2D is associated with increased morbidity and mortality due to its debilitating and progressive nature and associated complications. The condition usually leads to multiorgan failure due to macrovascular and microvascular involvement (Figure 1) [2–5].

Uncontrolled T2D can complicate pregnancy outcomes. Different kinds of birth defects are more commonly seen in babies born to women with diabetes [3]. Twin studies can estimate the multifactorial genetic involvement in T2D more precisely and have reported high degree of heritability of diabetes-related conditions such as disorders of first phase insulin response and basal and insulin-stimulated glucose uptake [6]. There are different methods for mapping the genetic susceptibility loci in the pathogenesis of T2D. Candidate gene studies and genome-wide studies are commonly used to identify the association of susceptible genetic loci of T2D. The latter includes both genome-wide linkage studies (GWL) and genome-wide association studies (GWAS) [6]. With the advent of recent molecular genetic techniques and rapid screening methods, the method of investigation has shifted to the use of molecular genetic markers for understanding the genetic aetiology of T2D and its complications [6, 7]. The common susceptible genetic variants are known to have a prominent effect on the risk of T2D across the world in multiple ethnic groups [8, 9]. Some variants appear to exert more pronounced genetic effects in specific ethnic groups. Most loci associated with T2D map to regulatory or intronic regions of the genome [9].

Diabetic peripheral neuropathy (DPN) is one of the debilitating microvascular complications of diabetes that is present in approximately 50% of patients. It is the primary cause of diabetes-related hospital admissions and nontraumatic foot amputations [4, 5, 10]. The molecular mechanisms involved in the development of DPN is a complex process that includes activation of the polyol pathway, exaggerated oxidative stress, overactivity of protein kinase C and increased formation of advanced glycation end-products in the presence of hyperglycemia. In addition, there is increasing evidence that genetic factors could also contribute to the development of DPN [10, 11]. The consequences of diabetic neuropathy include neurogenic pain, numbness, lack of coordination of voluntary movements, and a susceptibility to foot ulceration that leads to infections and toe or foot amputations. The rate of toe or foot amputations is 15 times greater in diabetic patients compared with individuals without diabetes. To date, approximately 80 T2D susceptibility genetic loci have been reported in different ethnic groups worldwide [12–14]. Majority of studies on the prevalence and associated aetiological factors of DPN have been conducted in Western countries. There is very limited data currently available for South Asian populations [15].

The objective of this paper is to provide an up-to-date review of the published scientific literature on the susceptible and prognostic genetic variants associated with DPN.

## 2. Methodology

This is a comprehensive review of the published literature on the susceptible and prognostic genetic variants associated with DPN. These variants were identified by an extensive survey of the scientific literature using the criteria described below. The most recent and relevant papers published in the last 15 years from January 2002 to July 2017 were searched in the PubMed database using the search terms “Diabetic peripheral neuropathy/genetics” and “genome-wide association study.” Altogether, sixty studies describing single nucleotide variants (SNVs) in genes associated with the susceptibility and prognosis of DPN which were published as full text articles in English during the defined period of study were included in this review. Epigenetic modifications which regulate gene expression mainly at the tissue/cellular level were excluded as it was outside the scope of this review.

## 3. Diabetic Peripheral Neuropathy

According to the Toronto Consensus Panel on Diabetic Neuropathy, DPN is defined as a symmetrical, length-dependent sensorimotor polyneuropathy that develops on a background of longstanding hyperglycemia, associated derangements, and cardiovascular risk factors [16]. The mechanisms underlying the pathogenesis of DPN are different between type 1 and type 2 diabetes mellitus [17]. Recent literature has reported that there are different groups of susceptible genetic loci which are clearly involved in the development of DPN. Different studies reported that some patients with prediabetes develop neuropathic complications, whereas others reported little evidence of neuropathy even after long-standing diabetes. This observation confirms the involvement of genetic aetiological factors associated with the development of DPN [18]. The data from different studies suggest that T2D and its complications may have shared genetic risk factors [12, 18].

## 4. Genetic Aetiology and Pathogenesis of DPN

The pathogenesis of DPN is a complex process and is involved with hyperglycemia-induced oxidative stress and altered polyol metabolism that changes the nerve microvasculature, growth factor support, and lipid metabolism [4]. It is important to identify these factors alone or in combination to arrange effective DPN treatment, as better understanding of the mechanisms underlying the onset and progression of DPN is of prime importance in the process of management [19]. Different groups of cell types in diabetic complication-prone tissues are targets of damage due to uncontrolled hyperglycemia. Schwann cells are the prime target of hyperglycemia which results in cell damage leading to altered axon integrity and defective growth factor signaling [20, 21]. Defective inflammatory pathways including advanced glycation end-product/receptor (*AGE/RAGE*) signaling in axons and Schwann cells have been reported in experimental animals with diabetic neuropathy which contributed to nerve damage [22].

Lu et al. in China studied SNVs from previously identified ten genetic loci and analyzed the association of these loci with peripheral nerve function in patients with T2D. They found that rs5219 of *KCNJ11* gene polymorphism (*E23K, G>A*) was associated with peripheral nerve function. The results obtained from nerve conduction studies (NCS) showed that the allele “A” had a protective effect on peripheral nerve function. They also reported that SNVs rs7756992 of *CDKAL1* and rs7903146 of *TCF7L2* were associated with DPN in the Chinese T2D population [23].

Yigit et al. identified 230 unrelated patients with DPN at the outpatient clinics of the Physical Therapy and Rehabilitation Department of Gaziosmanpasa University, Tokat, in Turkey. They investigated the distributions of the genotype and allele frequencies of the *MTHFR* gene C677T variant among patients with DPN and a matched control group. A statistically significant difference of *MTHFR* gene C677T polymorphism between the patients with DPN and the control group was identified [24].

Decreased levels of peroxisome proliferator-activated receptor alpha (*PPARA*) in chromosome 22 and lipid metabolism-related gene apolipoprotein E (*APOE*) in chromosome 19 have been identified confirming the findings that altered lipid metabolism may play a role in the progression of DPN [25]. Monastiriotis et al. reviewed the literature to identify the association between *APOE* polymorphism and DPN and found that the *ε*4 allele of the apolipoprotein E gene is significantly associated with the pathogenesis of DPN [25].

The alpha2B adrenergic receptor encoded by *ADRA2B* gene located on chromosome 2 is associated with an array of functions. A polymorphism (12Glu9) resulting in the insertion/deletion of three glutamic acid residues in the third intracellular loop has been described frequently in the literature [26]. In the nervous system, this polymorphism has been reported to be linked with autonomic nervous dysfunction. This is particularly increased with sympathetic nervous system activity, and Papanas et al. found a significant association in this indel allele distribution of alpha2B adrenoceptor gene among T2D patients with DPN in comparison with matched T2D patients without neuropathy [26].

## 5. Network of Genes Associated with Common Variants of DPN

**Figure 2:**
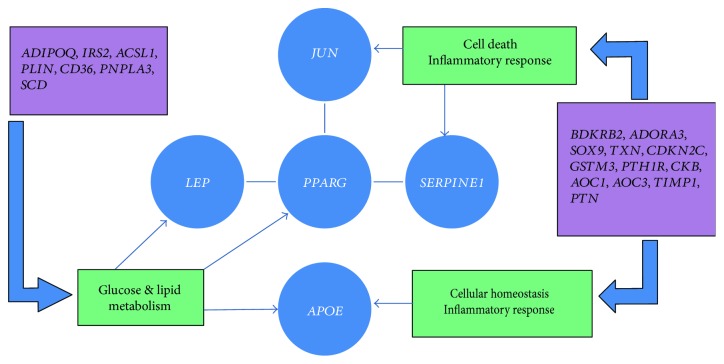
Five main genes (in blue circles) associated with diabetic peripheral neuropathy: *JUN*, *PPARG*, *LEP*, *SERPINE1*, and *APOE* and their relationship with defense response, inflammatory response, glucose, and lipid metabolism pathways (in green-coloured cages) are represented in the figure. Additional genes involved with DPN in relation to abovementioned metabolic pathways are indicated in purple-coloured cages. ADIPOQ = adiponectin, C1Q and collagen domain containing; IRS2 = insulin receptor substrate 2; ACSL1 = acyl-CoA synthetase long chain family member 1; PLIN = lipid storage droplet 2-like; CD36 = CD36 molecule; PNPLA3 = patatin-like phospholipase domain containing 3; SCD = stearoyl-CoA desaturase; BDKRB2 = bradykinin receptor B2; ADORA3 = adenosine A3 receptor; SOX9 = sex determining region Y-box 9; TXN = thioredoxin; CDKN2C = cyclin-dependent kinase inhibitor 2C; GSTM3 = glutathione S-transferase mu 3; PTH1R = parathyroid hormone 1 receptor; CKB = creatine kinase B; AOC1 = amine oxidase, copper containing 1; AOC3 = amine oxidase, copper containing 3; TIMP1 = TIMP metallopeptidase inhibitor 1; PTN = pleiotrophin.

Hur et al. examined two groups of DPN patients. A network of transcription factors jun (*JUN*), leptin (*LEP*), serpin peptidase inhibitor E type 1 (*SERPINE1*), apolipoprotein E (*APOE*), and peroxisome proliferator-activated receptor gamma (*PPARG*) was examined to identify their potential relationship (Figure 2). Further subsets of genes related to defense response, inflammatory response, regulation of lipid metabolic processes, and *PPAR* signaling pathways were then analyzed to identify the association of gene expression and development of DPN (Figure 2) [27]. They demonstrated that increased glucose metabolism due to hyperglycemia resulted in increased oxidative stress, mitochondrial dysfunction, and cell death in both in vitro and in vivo models of diabetic neuropathy [27].

## 6. Variants Associated with Defense Response and Inflammatory Response in the Pathogenesis of DPN

Hur et al. reported that the molecules which are involved with the process of inflammation such as chemotactic agents and cytokines are involved with the development and progression of DPN as well as diabetic nephropathy [27, 28]. Kakoki et al. identified that the bradykinin receptor B2 (*BDKRB2*) is of particular interest in disease progression of DPN. *BDKRB2* gene was found to be involved in progressive glomerulosclerosis and also susceptibility to DPN [29].

Membrane-associated adenosine A3 receptor (*ADORA3*) is also involved in the pathogenesis of DPN [30]. Variants of *BDKRB2* and *ADORA3* were found to be involved in enhanced inflammation and dysregulated defense responses, thus contributing to more substantial nerve damage in patients with progressive DPN [27, 30]. Gene variants of *TXN*, *CDKN2C*, *GSTM3*, *PTH1R*, *CKB*, *AOC1*, *AOC3*, *TIMP1*, and *PTN* have been identified as other additional genes associated with defense response and inflammatory response in the pathogenesis of DPN (Figure 2) [27].

## 7. Variants Associated with Glucose Metabolic Processes and PPAR Signaling Pathway in the Pathogenesis of DPN

According to Hur et al., *PPARG*, which encodes a nuclear receptor for glitazone, plays a key role in regulating glucose and lipid metabolism [27, 31]. Agonists of *PPARG* are effective in treatment of DPN and nephropathy in experimental animal models [27, 32]. Another key gene is *APOE*, encoding an apolipoprotein, which regulates the normal catabolism of triglycerides and cholesterol. A polymorphism of this gene is linked to the progression of DPN [33]. Gene variants of *ADIPOQ*, *IRS2*, *ACSL1*, *PLIN*, *CD36*, *PNPLA3*, and *SCD* were identified as other additional gene variants associated with glucose metabolic processes and PPAR signaling pathway in the pathogenesis of DPN (Figure 2) [27].

## 8. Genetic Variants Involved in Different Phenotypes of DPN

According to Cheng et al., in an experiment involving both human and animal models, sensory neurodegeneration in the chronic stage of diabetes was found to be associated with early damage to the distal axons of both upper and lower limb neurons showing a pattern that accounts for the distribution of “glove-and-stocking” loss of sensation characteristically seen in DPN. These changes accompany widespread abnormalities involving electrophysiology and alterations in gene expression that indicate a degenerative phenotype. However, existing knowledge on the development of DPN which includes oxidative and nitrergic stress, polyol accumulation, microangiopathy, inappropriate *AGE-RAGE* signaling, and/or mitochondrial dysfunction account for diverse mRNA changes that alter miRNA expression patterns resulting in diverse DPN phenotypes [34].

## 9. Genetic Variants Involved in Gender Dimorphism of DPN

Significant gender dimorphisms in the responsiveness of patients to antidiabetic drugs have been reported in the literature [35–37]. These observations highlighted the importance of understanding the gender-specific differences in manifestation of diabetes mellitus and its complications such as DPN.

O'Brien et al. reported the first instance of a female T2D mouse model presenting with a neuropathic phenotype including decreased intraepidermal nerve fiber density, impaired motor and sensory nerve conduction velocities, and thermal hypoalgesia [38]. A GWAS involving 961 diabetic neuropathic pain cases and 3260 diabetic controls in the Genetics of Diabetes Audit and Research Tayside by Meng et al. found that a cluster in the 1p35.1 region, the zinc finger and *SCAN* domain containing 20 (*ZSCAN20*) with a lowest *p* value of a variant at rs71647933 in females, and a cluster in the 8p23.1 region next to *HMGB1P46* with a lowest *p* value of a variant at rs6986153 in males were significantly associated with DPN. This GWAS on diabetic neuropathic pain provides evidence for the sex-specific involvement of 1p35.1 region (*ZSCAN20*) and 8p23.1 region (*HMGB1P46*) [39].

## 10. Other Gene Loci Involved in the Pathogenesis and Prognosis of DPN

A fibrinolysis-regulating gene, *SERPINE1*, which encodes for plasminogen activator inhibitor 1 (*PAI-1*) has been identified in association with higher incidences of diabetic complications such as diabetic neuropathy and nephropathy in knockout *PAI-1* mice [40, 41]. The cell cycle controlling *JUN* is also involved in the progression of DPN and is associated with inflammation and insulin resistance which is activated in multiple tissues including the peripheral sensory nerves of patients with types 1 and 2 diabetes [42].

Three other subsets of important gene variants were documented in literature associated with “cell projection and axonogenesis” involving nerve growth factor receptor (*NGFR*) and “cellular homeostasis and inflammatory response” involving thioredoxin, and “cytoskeletal protein binding” with stathmin 1 (*STMN1*) genes. *NGFR* exhibits protection against nerve cell and axonal damage, and the expression of nerve growth factor receptor protein in plasma correlates with DPN progression in diabetic rat models [43]. Thioredoxin, which regulates cellular oxidative stress with its antioxidant activity, also plays an important role in associated diabetes. Thioredoxin's antioxidant activity is significantly inhibited by hyperglycemic states in the blood. It complicates diabetes by playing an important role by deregulating vascular oxidative stress and inflammation in diabetic patients [44].

A study in North Catalonia, Spain, by Jurado et al. identified the protective effect of a single angiotensin-converting enzyme (*ACE*) gene polymorphism on the development of DPN in T2D patients. Despite *ACE* gene variants which are associated with diabetic renal disease and/or diabetic retinopathy, the heterozygous genotype stands as a protective factor against the development of DPN [45]. Heterozygous (D/I) *ACE* gene polymorphism reported a statistically significantly reduced risk of developing DPN whereas homozygous (D/D) *ACE* gene polymorphism reported an increased risk [45].

Mitochondrial transcription factor A (*TFAM*) is located in mitochondria, and its level regulates mitochondrial DNA (mtDNA) copy number. Chandrasekaran et al. showed that *TFAM* over expression prevented a decrease in mtDNA copy number in diabetic dorsal root ganglia (DRG) neurons, helped prevent DPN, and protected DRG neurons from oxidative stress in experimental mouse models [46].

Aldo-keto reductase family 1 member B (*AKR1B1*) in chromosome 7 encodes a member of the aldo-keto reductase superfamily, which consists of more than 40 known enzymes and proteins. This catalyzes the reduction of a number of aldehydes and is thereby implicated in the development of diabetic complications by catalyzing the reduction of glucose to sorbitol. Saraswathy et al. identified significant association of *AKR1B1* gene mutations in painful diabetic neuropathy [47].

There is increasing evidence that microRNAs (miRNAs) act as regulators of gene expression in multiple biological processes and associated complications [48]. Ciccacci et al. looked for an association between variants in miRNA genes and DPN. The results of this study identified a role for *MIR146a* and *MIR128a* SNVs in the susceptibility to DPN and were shown to have a significant association [49]. The rs2910164 (G>C) in *MIR146a* is associated with lower risk, and rs11888095 (C>T) in *MIR128a* is associated with higher risk of susceptibility to DPN [49].

Nitric oxide (NO) production and local release in the tissues significantly contributed to endothelial dysfunction. The process takes place by the modulation of the nitric oxide synthase (NOS) enzymes responsible for NO synthesis. Endothelium-derived NO plays a key role in the regulation of vascular tone and has vasoprotective effects by removing superoxide radicals and suppressing platelet aggregation, leukocyte adhesion, and smooth muscle cell proliferation. However, dysfunctional endothelial nitric-oxide synthase (eNOS) might play a critical role in the pathogenic pathway leading to diabetic vascular complications including DNP. Therefore, eNOS is considered as a candidate for the progression of DPN [50, 51].

In the early stages of DPN, abnormalities in the vasa nervorum and loss of nerve fibers can be seen in association with hyperglycemia. Damage to the nervous tissue results in increased intravascular endothelial growth factor (*VEGF*) plasma levels in diabetic animal models [52]. The ischemia and hypoxia in the nerves of patients with T2D due to microangiopathy of vasa nervorum have always been observed and may be a key pathogenic mechanism of DPN [53]. An association study by Ghisleni et al. showed a clear association between diabetic polyneuropathy and the C936T polymorphism of the *VEGF* gene and the C242T polymorphism of the *p22phox* allele of *CYBA* gene [52].

Functional *GRP78* variants in heat shock protein family A (Hsp70) member 5 (*HSPA5*) genes are likely to have some influence on the gene expression, which results in the dysfunction of peripheral nerves and neuropathy. According to Jia et al., functional *GRP78* rs391957 variants, which are located in the promoter region, 57168556T>C, are known to cause abnormal promoter activities significantly associated with DPN [54].

Adiponectin gene (*ADPN*) serves as a protective factor in preventing diabetes progression by suppressing inflammatory responses and increasing insulin sensitivity [55]. SNVs of *ADPN* may influence T2DM, but *ADPN* variants SNV45 (45T/G, rs2241766) and SNV276 (276G/T, rs1501299) are the two most prominent variants influencing the disease progression, especially pathogenesis of DPN [56, 57]. A case-control study conducted by Ji et al. to evaluate the association between *ADPN* gene variants and pathogenesis of DPN in T2D patients indicated an increased risk of DPN in T2D patients, by downregulating *ADPN* expression which resulted in significantly reduced circulating *ADPN* plasma levels. Furthermore, they reported that the polymorphism frequencies of GG and GT haplotypes in the DPN group were significantly lower than those in the matched control group, while the frequency of the TG haplotype in the DPN group was markedly higher than that in the control group, showing a clear association between *ADPN* gene variants and the risk of DPN [58].

## 11. Genetic Variants of DPN in Different Ethnic Populations

Up to now, only few ethnic groups have described population-specific genetic variants associated with DPN. In a study conducted by Lu et al., 10 SNVs associated with pathogenesis of T2DM were studied. They reported that rs5219 on *KCNJ11* (E23K) gene is significantly associated with peripheral nerve function in a Chinese population with T2D [23]. Jia et al. studied the significance of functional *GRP78* gene variants in predicting the onset of type 2 DPN in the Chinese population. They suggested that the *GRP78* rs391957 promoter polymorphism is a potential risk factor for type 2 DPN in this population [54]. Prasad et al. studied forty-two patients with T2D from the Institute of Diabetology, Madras Medical College, and Rajiv Gandhi Government General Hospital in Chennai, Tamil Nadu, India. In this study, the extent of DNA damage in patients suffering from T2D, both with and without neuropathy, was analyzed. No genetic variants were evaluated in this study. The data demonstrated that the frequency of DNA damage was significantly higher in the T2D patients with DPN than in the controls [59]. Stoian et al. conducted a study in the University Center of Tırgu Mures, Romania. In their case-control study, which included a total of 182 participants, including 84 unrelated patients with T2D and an age-matched control group consisting of 98 unrelated individuals without T2D, they evaluated the influence of *GSTM1*, *GSTT1*, and *GSTP1* variants on T2D and DPN risk. Their data suggested that *GSTM1*, *GSTT1*, and *GSTP1* gene variants were not associated with individual susceptibility to developing DPN in patients with T2D in the Romanian population [60].

An association study of C936T polymorphism of the *VEGF* gene and the C242T polymorphism of the *p22phox* gene with T2D and DPN in a population of Caucasian ethnicity was studied by Ghisleni et al. According to their results, the C936T polymorphism of the *VEGF* gene and C242T polymorphism of the *p22phox* gene did not correlate with the risk of developing diabetes mellitus or neuropathic signs and symptoms. When considering the results of other studies, a substantial heterogeneity in the findings is observed, which demonstrates a complex link between the risk factors of DM and genetic predisposition to DPN [52]. Common susceptible and prognostic genetic factors associated with DPN in T2D are diverse in different pathophysiological pathways, and it is difficult to separate each genetic variant from the other as most of the variants are interrelated with each other (Table 1).

## 12. Conclusions

Although targeted gene sequencing is still a method of choice to identify rare functional mutations in monogenic disorders, exome sequencing becomes an attractive and cost-effective alternative when other disease-mapping strategies provide few or ambiguous results. This review has attempted to identify the common susceptibility and prognostic genetic factors associated with DPN in T2D. Knowledge about these factors is vital as DPN is one of the debilitating complications associated with T2D and identification of the common genetic variants would be valuable for the future development of gene panels targeted for the early detection and prognosis of DPN. Together with these gene panels, further gene expression studies will need to be conducted to modulate effective targeted therapies for DPN in these patients.

## Figures and Tables

**Table 1:** Genetic variants associated with DPN.

Gene	Chromosomal location	Variants	Associated risk/remarks	Reference
Advanced glycation end receptor (*AGER*)	6p21.32	rs1800624	Higher risk/defective inflammatory pathways	[22]
Peroxisome proliferator-activated receptor alpha (*PPARA*)	3p25.2	rs1801282	Higher risk/defective inflammatory pathways	[27]
Bradykinin receptor B2 (*BDKRB2*)	14q32.2	rs1799722	Higher risk/defective inflammatory pathways/African-Americans	[29]
Potassium voltage-gated channel subfamily J member 11 (*KCNJ11*)	11p15.1	E23K, G>A rs5219	Higher risk/Chinese population/altered signaling pathways	[23]
CDK5 regulatory subunit-associated protein 1-like 1 (*CDKAL1*)	6p22.3	rs7756992	Higher risk/Chinese population	[23]
Transcription factor 7-like 2 (*TCF7L2*)	10q25.2-q25.3	rs7903146	Higher risk/Chinese population	[23]
Methylenetetrahydro folate reductase (*MTHFR*)	1p36.22	C677T rs1801133	Higher risk/altered folate metabolism	[24]
Apolipoprotein E (*APOE*)	19q13.32	*ε*4 allele- rs429358 rs7412	Higher risk/altered lipid metabolism	[25]
Adrenoceptor alpha 2B (*ADRA2B*)	2q11.2	12Glu9 rs879255577	Higher risk/defects in regulation of neurotransmitter release from sympathetic nerves	[26]
microRNA 146a (*MIR146a*)	5q33.3	rs2910164 (G>C)	Lower risk	[49]
microRNA128a (*MIR128a*)	2q21.3	rs11888095 (C>T)	Higher risk	[49]
High mobility group box 1 pseudogene 46 (*HMGB1P46*)	8q23.1	rs6986153	Males/higher risk	[39]
Zinc finger and SCAN domain containing 20 (*ZSCAN20*)	1p35.1	rs71647933	Females/higher risk	[39]
Serpin family E member 1 (*SERPINE1*)	7q22.1	rs1799768	Progressive type of DPN	[40]
Nerve growth factor receptor (*NGFR*)	17q21.33	rs734194	Progressive type of DPN	[43]
Angiotensin-converting enzyme (*ACE)*	17q23.3	rs1799752 diallelic polymorphism: presence/absence of 287bp in intron 16 Heterozygous D/I Homozygous D/D	Japanese population Lower risk Higher risk	[45]
Aldo-keto reductase family 1 member B (*AKR1B1*)	7q33	rs5053 rs759853	Higher risk/altered glucose metabolism	[47]
Vascular endothelial growth factor (*VEGF*)	6p21.1	C936T/rs3025039 rs2010963 rs699947	Higher risk	[52]
Cytochrome b-245 alpha chain (*CYBA*)	16q24.2	C242T rs4673	Higher risk	[52]
Heat shock protein family A (Hsp70) member 5 (*HSPA5*)	9q33.3	Promoter region 57168556T>C rs391957	Higher risk	[54]
Adiponectin (*ADIPOQ*)	3q27.3	45T/G rs2241766	Higher risk	[56, 57]
		276G/T rs1501299	Higher risk	
